# Xigua Video as a Source of Information on Breast Cancer: Content Analysis

**DOI:** 10.2196/19668

**Published:** 2020-09-29

**Authors:** Peng Pan, Changhua Yu, Tao Li, Xilei Zhou, Tingting Dai, Hanhan Tian, Yaozu Xiong

**Affiliations:** 1 Department of Radiation Oncology The Affiliated Huaian No.1 People's Hospital of Nanjing Medical University Jiangsu province Huaian China

**Keywords:** breast cancer, internet, Xigua Video, content analysis

## Abstract

**Background:**

Seeking health information on the internet is a popular trend. Xigua Video, a short video platform in China, ranks among the most accessed websites in the country and hosts an increasing number of videos with medical information. However, the nature of these videos is frequently unscientific, misleading, or even harmful.

**Objective:**

Little is known about Xigua Video as a source of information on breast cancer. Thus, the study aimed to investigate the contents, quality, and reliability of breast cancer–related content on Xigua Video.

**Methods:**

On February 4, 2020, a Xigua Video search was performed using the keyword “breast cancer.” Videos were categorized by 2 doctors based on whether the video content provided useful or misleading information. Furthermore, the reliability and quality of the videos were assessed using the 5-point DISCERN tool and 5-point global quality score criteria.

**Results:**

Out of the 170 videos selected for the study, 64 (37.6%) were classified as useful, whereas 106 (62.4%) provided misleading information. A total of 41.8% videos (71/170) were generated by individuals compared to 19.4% videos (33/170) contributed by health care professionals. The topics mainly covered etiology, anatomy, symptoms, preventions, treatments, and prognosis. The top topic was “treatments” (119/170, 70%). The reliability scores and global quality scores of the videos in the useful information group were high (*P*<.001). No differences were observed between the 2 groups in terms of video length, duration in months, and comments. The number of total views was higher for the misleading information group (819,478.5 vs 647,940) but did not reach a level of statistical significance (*P*=.112). The uploading sources of the videos were mainly health care professionals, health information websites, medical advertisements, and individuals. Statistical differences were found between the uploading source groups in terms of reliability scores and global quality scores (*P*<.001). In terms of total views, video length, duration, and comments, no statistical differences were indicated among the said groups. However, a statistical difference was noted between the useful and misleading information video groups with respect to the uploading sources (*P*<.001).

**Conclusions:**

A large number of Xigua videos pertaining to breast cancer contain misleading information. There is a need for accurate health information to be provided on Xigua Video and other social media; health care professionals should address this challenge.

## Introduction

Cancer incidence and mortality have been increasing in China, which have raised major health concerns [1]. Breast cancer ranks fifth among newly diagnosed cancers and is the most common form among Chinese women [2].

By the end of June 2019, the number of internet users in China had reached 854 million, out of which mobile internet users accounted for 99.1% of the total netizen population. Moreover, 759 million online video users accounted for 88.8% of the total internet users [3]. With the increasing popularity of online information, people tend to use the internet as an important source of health care information. Previous studies have reported that 80% of internet users access health information online [4], as freely available video websites are popular channels of information dissemination.

Xigua Video is a short video platform of aggregative media Jinri Toutiao (“today’s headlines”) run by Beijing ByteDance Technology Co Ltd. It is estimated to be the largest site for sharing of short videos in China [5]. Xigua Video places emphasis on user-uploaded videos and has accumulated an average of 120 million daily views [6]. Although information from the website is easily accessible, users may be unable to judge the quality and accuracy of its contents [7-10].

Previous studies have examined the quality of online information on various health topics [11-19]. Unfortunately, many studies revealed poor-quality, biased, and commercial content, which may lead to dangerous consequences for users.

To date, data on the evaluation of Xigua Video as a source of health information remain lacking. To better understand how Chinese internet users discuss breast cancer on social media platforms such as Xigua Video, we conducted an analysis to evaluate the content, quality, and reliability of Chinese-language breast cancer–related information available on Xigua Video.

## Methods

### Data Collection

On February 4, 2020, a Xigua Video search was performed using the keyword “breast cancer” for videos. All videos uploaded up by the search date were included. In the case of duplicate videos, only 1 was taken into consideration. Videos were excluded if they were irrelevant or lacked accompanying audio. As a result, the study obtained 170 videos in total.

After collecting data on content, duration in months on Xigua Video, number of views, length, and comments, 2 physicians independently evaluated the overall quality of the videos. The kappa statistic for the 2 reviewers was κ=0.78, which indicated substantial agreement [20].

### Creation of Content Categories

As the title often does not reflect the actual content of the video, the study considered topics for categorization. Content was categorized according to a previous YouTube study on colorectal cancer [21]. If a video covered more than 1 topic, then each topic was listed separately.

In addition, the videos were classified into 4 groups according to source, namely, health care professionals, health information websites, medical advertisements, and individuals.

### Scoring and Classification of Videos

Videos were classified into 2 groups, namely, useful information and misleading information. Videos rated as useful contained scientifically accurate information about any aspect of breast cancer, such as symptoms, treatment, and prevention. Conversely, misleading videos contained inaccurate or unproven information about the disease. This classification method has been used in other studies [22,23].

Video reliability was scored using the 5-point DISCERN tool [24]. In addition, the overall quality of the videos was rated using the 5-point global quality score criteria [25]. In both instruments, higher scores indicate higher content quality of the video clip.

### Statistical Analysis

Statistical analysis was performed using SPSS 22.0 (IMB Corp). Numerical variables were reported as mean (SD) or median (IQR) values. The Student *t* test, the Mann-Whitney *U* test, analysis of variance, and the Kruskal-Wallis test were applied for the comparison of numerical variables. The Dwass-Steel-Critchlow-Fligner post hoc test was conducted after the Kruskal-Wallis test. Categorical variables were stated as number (n) and percentage (%). In the comparison of categorical variables, chi-square and Fisher exact tests were used. A value of *P*<.05 was considered statistically significant.

### Ethical Approval and Consent to Participate

This study did not require approval by the local research ethics board since only publicly available data were used.

## Results

### Topics and Uploading Sources

The videos were analyzed based on the topics covered therein. In all categories, the topic of treatments was the most frequently covered (119/170, 70%), followed by symptoms (56/170, 33%), prognosis (44/170, 26%), anatomy (34/170, 20%), prevention (25/170, 15%), and etiology (18/170, 11%).

A total of 41.8% (71/170) of the videos were posted on the website by individuals. Medical advertisements and health information websites were responsible for uploading 25.9% (44/170) and 12.9% (22/170) of the total videos, respectively. The videos contributed by health care professionals accounted for only 19.4% (33/170).

### Information Reliability and Quality

As previously mentioned, the selected videos were classified into useful and misleading groups. Of the 170 selected videos, the number of videos containing misleading information was 106 (62.4%); 64 (37.6%) contained useful information. Table 1 presents the classification of the video characteristics. A statistically significant difference was determined in the useful information group with respect to the reliability and global quality scores (*P*<.001). However, no significant differences were noted in terms of total views (*P*=.11), video length (*P*=.66), duration in months on Xigua Video (*P*=.051), and comments between the 2 groups (*P*=.73).

In addition, videos were compared according to their ownership. Table 2 presents the analysis of the video characteristics by uploading sources. Statistically distinctive differences between the uploading sources were observed for the reliability and global quality scores (*P*<.001). In terms of total views (*P*=.22), video length (*P*=.06), duration in months (*P*=.15), and comments (*P*=.47), no statistical differences were found. In the useful information group, 35.9% (23/64) and 20.3% (13/64) of the videos were generated by health care professionals and health information websites, respectively. On the contrary, 50% (53/106) of the videos in the misleading information group were generated by individuals. Data indicated statistical difference between the useful and misleading groups in terms of uploading sources (*P*<.001).

**Table 1:** Analysis of video characteristics by usefulness category.

Characteristics	Useful group	Misleading group	*P* value
Views, IQR	592,515.25	894,786	.11
Video length (s), IQR	234	264.5	.66
Duration in months on Xigua Video, mean (SD)	11.9 (7.6)	10.7 (5.9)	.051
Comments, IQR	37	42.5	.73
Reliability score, mean (SD)	2.34 (1.03)	1.45 (1.01)	<.001
Global quality score, mean (SD)	3.58 (1.24)	2.12 (0.99)	<.001

**Table 2:** Analysis of video characteristics by uploading sources.

Characteristics	Health care professionals	Health information websites	Medical advertisements	Individuals	*P* value
Videos (N=170), n (%)	33 (19.4)	22 (12.9)	44 (25.9)	71 (41.8)	N/A^a^
Reliability score, mean (SD)	2.85 (1.12)	2.55 (1.26)	2.07 (0.82)	1.65 (1.11)	<.001
Global quality score, mean (SD)	3.45 (1.06)	3.50 (1.34)	1.89 (0.92)	2.37 (1.123)	<.001
Total views, IQR	1,010,639	840,070.75	898,145	857,950	.22
Video length (s), IQR	246	245	209	260	.06
Duration on Xigua Video (months), mean (SD)	13.5 (8.1)	10.0 (7.1)	10.6 (5.6)	10.7 (6.1)	.15
Comments, IQR	36.5	62.3	46.8	41	.47
Misleading information (n=106), n (%)	10 (9.4)	9 (8.5)	34 (32.1)	53 (50.0)	<.001
Useful information (n=64), n (%)	23 (35.9)	13 (20.3)	10 (15.6)	18 (28.2)	N/A

^a^N/A: not applicable.

## Discussion

### Overview

Breast cancer is the most common form of cancer and accounts for 30.4% of total cancer incidence and 2.51% of total mortality due to cancer among women in China. Notably, the incidence rate of breast cancer has increased rapidly in women aged 20-55 years. Therefore, the effectiveness of strategies for prevention, early detection, and management of breast cancer is an essential aspect to be considered.

A broad range of information is available on the internet. The China Research Institute for Science Popularization analyzed netizens' behavior and requirements for information [26]. The results indicated that the science communication search index, which is based on the amount of time people spend on search engines, increased from 2.8 billion in 2014 to 9.2 billion in 2019. Young and middle-aged netizens were the main drivers of the trend with health and medical care as the top topic. With the rapid development of the internet, new media are providing important access to health and medical information. Previous studies reported YouTube as an information source for various forms of cancers [27-31]. Although YouTube is inaccessible in China for a number of reasons, many similar video websites deliver the same functionality, such as Jinri Toutiao, which was launched in 2012. The website has gained 200 million monthly active users [6]. Specifically, Xigua Video is the video platform of Jinri Toutiao. To the best of our knowledge, no study has assessed the contents, quality, and reliability of videos on the internet as a source of health information related to breast cancer in China.

Restricted by relevant Chinese laws, users’ gender and age cannot be displayed. However, the study obtained useful information from other sources. According to the Baidu search engine’s statistics on “breast cancer” search results, approximately two-thirds of users are women, and 90% are aged 20-50 years. Therefore, we infer that Jinri Toutiao users who search for information on breast cancer are mainly young and middle-aged female netizens.

### Principal Findings

Our study found that nearly half of the videos generated by individuals covered the topic of treatment. Alarmingly, approximately two-thirds of the videos spread misleading information. Furthermore, the quality of the videos was low. In addition, the fact that viewers gave better ratings to poor-quality videos compared to the high-quality videos indicates that the majority of health seekers were incapable of recognizing low-quality medical information in videos.

### Low Percentage of Good- or Excellent-Quality Videos

The topics of the videos observed in the study mainly covered etiology, anatomy, symptoms, prevention, treatments, and prognosis. The majority of the videos only contained 1 or 2 of the above-mentioned categories. In all the categories, treatments ranked first. The videos examined gained a total of 13 million views with a total watch time of more than 16 hours and 8598 comments. These data illustrate that videos on breast cancer are extremely popular. However, the contents of many videos frequently lack peer review, which limits the reliability and accuracy of the videos on Xigua Video. Although the reliability and global quality scores of the useful videos were high, the number of useful videos (64/170) was lower than that of the misleading videos (106/170). Notably, the number of total views was higher for the videos in the misleading information group (IQR 894-786) than that for the useful information group (IQR 592-515.25). These findings indicate that viewers may find it difficult to differentiate between useful and misleading information. A total of 41.8% (71/170) videos were produced by individuals, the majority (53/71, 74.6%) of which contained misleading information. Given that users who want to access information are mostly non–health care personnel, results indicate obtaining accurate information from Xigua videos is difficult.

In general, more reliable information can be obtained from videos uploaded by health care professionals. However, the proportion of these videos reached only 35.9% (23/64) in the useful information group. In other words, only one-third of useful videos had been uploaded by health care professionals. This result is similar to that reported by Mueller et al [4].

The most commonly watched videos were those containing misleading information, whereas the least watched videos were those from health care professionals. These results confirm that effective measures should be taken to reduce inaccurate (and possibly harmful) information on this video platform.

The results further indicated that videos from health care professionals have significantly higher reliability and global quality scores than those posted by individuals. As such, lay users find difficulty in distinguishing useful information from a massive number of videos. Moreover, the results indicated that ownership is an important element that can be used to assess the reliability of videos. If professionals such as doctors, health care organizations, and health information websites produced videos, then the content of those videos may be considered trustworthy [32]. The study demonstrated that videos produced by health care professionals were geared toward more educational purposes. This finding is in accord with those of other studies [33-35]. Hence, such professionals should utilize their expertise and contribute high-quality videos for patients as information sources on websites. Conversely, individuals contributed 18 videos (28.2%) to the useful information group. However, our results emphasizes that these videos may not be able to cover all aspects of breast cancer. Therefore, many videos can contain some valuable information despite providing overall misleading content.

### Limitations

A limitation of this study is that only videos in the Chinese language were examined. Second, the results comprise a snapshot of information distribution to illustrate the quality of videos on the Xigua platform at one point in time. Therefore, these results may change as videos are added or removed over time.

### Conclusions

Despite the rising incidence of breast cancer, the level of public awareness in China remains low. Chinese websites, such as Xigua Video, provide a new medium for disseminating cancer information to the public through videos. For the sake of public awareness, health care and medical professionals should adopt this technology and take effective actions to provide accurate information about breast cancer on these video websites. The lack of reliable and useful health information on the internet remains a significant problem, and health care professionals and governments urgently need to address this challenge.
